# Supplementary material for: Increased Incidence and Clinical Features of Septic Arthritis in Patients Aged 80 Years and above: A Comparative Analysis with Younger Cohorts
Source: Pathogens. 2024 Oct 11;13(10):891. doi: 10.3390/pathogens13100891 (PMC11510638; doi:10.3390/pathogens13100891)
Supplement: Supplementary file 1 [file pathogens-13-00891-s001.zip › pathogens-3186103-supplementary.pdf]

Supplementary Table S1. Septic arthritis with isolated organism between 2016 through 2019. Comparison between two pathogenetic subgroups: direct inoculation group[a] and hematogenous group.

	18–64 years	65–79 years	80 years and above
Septic arthritis due to direct inoculation (*n* = 54) number (%)	36 (67)	11 (20)	7 (13)
Hematogenous septic arthritis (*n* = 216) number (%)	78 (36)	85 (39)	53 (25)

[a] Postoperative within 3 months, septic arthritis one to two weeks after intraarticular injection, trauma or bite at the affected joint.

Supplementary Table S2. Demographics and comparison between group of patients where SF-WBC was analysed and group of patients where SF-WBC was missing.

Parameter	SF-WBC is analysed (*n* = 74)	SF-WBC is missing (*n* = 142)	*p*-value
Male (%)	41 (55)	84 (59)	NS
Hospitalization days median (range)	19 (1–80)	16 (1–67)	NS
Mortality within 30 days (%)	3 (4)	8 (6)	NS
Osteosynthesis or prosthesis in affected joint	12 (16)	17 (12)	NS
Monoarthritis (%)	71 (96)	125 (88)	NS
Fever ≥ 37.5 Celsius (%)	43 (58)	71 (50)	NS
CRP mg/L mean (SD)	207 (119)	230 (134)	NS
Haemoglobin g/L mean (SD) *n* = 201	121 (19)	123 (20)	NS
WBC $\times 10^9$/L mean (SD)	15 (19)	15 (17)	NS
Platelets $\times 10^9$/L mean (SD)	280 (152)	239 (117)	NS
Creatinine µmol/L mean (SD)	126 (123)	128 (106)	NS

Supplementary Table S3. Comparison between demographics and characteristics of patients in the youngest age group, 18-64 years, divided into two new groups in order to analyze potential differences.

Parameter	18–30 years (*n* = 8)	31–64 years (*n* = 70)	*p*-value
Male (%)	5 (63)	39 (56)	NS
Hospitalization days median (range)	6 (1–14)	14 (2–80)	0.008
Mortality within 30 days (%)	0 (0)	0 (0)	NS
Fever ≥ 37.5 Celsius (%)	*n* = 7<br>4 (57)	*n* = 68<br>41 (60)	NS
CRP mg/L mean (SD)	74 (47)	209 (137)	0.004
Haemoglobin g/L mean (SD) *n* = 201	131 (16)	127 (19)	NS
WBC x10^9/L mean (SD)	*n*=7<br>12 (5)	*n*=67<br>13 (7)	NS
Platelets x10^9/L mean (SD)	*n* = 6<br>283 (164)	*n* = 60<br>278 (138)	NS
Creatinine µmol/L mean (SD)	*n* = 7<br>77 (17)	*n* = 62<br>112 (110)	NS
SF-WBC x10^9/L mean (SD)	*n* = 4	*n* = 21	NS

	53 (55)	42 (30)	
Glucose ratio mmol/L mean (SD)	*n* = 3<br>0.33 (0.52)	*n* = 21<br>0.39 (0.29)	NS
SF-PMN/WBC ratio mean (SD)	*n* = 4<br>0.85 (0.03)	*n* = 20<br>0.88 (0.12)	NS
Obesity	0 (0)	14 (20)	NS

[a] Obesity is defined as BMI >30. Mann-Whitney U was used for mean and median. Fishers exact test was used for statistics on sex, mortality within 30 days, fever and obesity. NS; not significant.
